# Assessing the Porosity-Binder Ratio and Machine Learning Models for Predicting the Strength and Durability of Soil-Cement-Glass Powder Geomaterial

**DOI:** 10.3390/ma19040823

**Published:** 2026-02-21

**Authors:** Jair Arrieta Baldovino, Oscar E. Coronado-Hernández, Yamid E. Nuñez de la Rosa

**Affiliations:** 1 Department of Civil Engineering, Universidad de Cartagena, Cartagena de Indias 130015, Colombia; 2 Instituto de Hidráulica y Saneamiento Ambiental, Universidad de Cartagena, Cartagena de Indias 130001, Colombia; ocoronadoh@unicartagena.edu.co; 3 Faculty of Engineering and Basic Sciences, Fundación Universitaria Los Libertadores, Bogotá 110231, Colombia

**Keywords:** ground glass powder, machine learning, porosity-cement index, ground improvement

## Abstract

This study evaluates the mechanical behavior and durability of a silty soil stabilized with Portland cement and recycled ground glass powder (*GGP*). The porosity–cement index (η/C_iv_) was applied to predict unconfined compressive strength ($q_{u}$), splitting tensile strength ($q_{t}$), and accumulated mass loss (*ALM*) under wetting–drying cycles. Mixtures were prepared with cement contents of 3%, 6%, and 9%, GGP contents of 5%, 15%, and 30%, and dry unit weights of 13.5, 14.5, and 15.5 kN/m^3^, and were cured for 7, 28, and 90 days. The experimental program consisted of a large dataset, comprising 486 mechanical tests (unconfined compressive and splitting tensile strength) and 81 durability tests, providing a robust basis for both empirical modeling and machine learning analysis. The results confirmed a strong power-law relationship between η/*C*_iv_ and both $q_{u}$ and $q_{t}$, achieving high coefficients of determination (*R*^2^ > 0.98). The strength coefficient (A) increased consistently with curing time and *GGP* addition, indicating enhanced pozzolanic reactivity and matrix densification. After 90 days, $q_{u}$ increased by over 250% and $q_{t}$ by nearly 700%. Durability tests revealed exponential reductions in *ALM* with higher density and binder content, achieving values below 0.5% for the densest mixtures, which contained 30% *GGP*. These findings validate the η/*C*_iv_ index as an effective predictor of strength and durability in soil–cement–*GGP* geomaterials, establishing a solid basis for future integration with machine learning models. The implementation of twenty-eight machine learning presets for predicting $q_{u}$, $q_{t}$, and *ALM* demonstrated that the Matern 5/2 Gaussian Process Regression and the trilayered neural network are the most suitable algorithms, achieving R^2^ values higher than 0.987 in both the validation and testing stages.

## 1. Introduction

### 1.1. Porosity-to-Binder Index in Soil Stabilization

Since 2007, the porosity–cement ratio has been widely used to estimate the strength, durability, and stiffness of various stabilized soils, including those treated with lime and cement, as well as those incorporating different waste materials and alternative binders. The porosity–cement ratio exhibits a close correlation with both the intrinsic properties of the soil and the characteristics of the binders employed [1]. This approach has been applied to evaluate the geotechnical properties of clean sands, silts, and clays. The empirical estimation of unconfined compressive strength ($q_{u}$) and splitting tensile strength ($q_{t}$) is often expressed in the following form (Equation (1)):

(1) $$q_{u}\;or\;q_{t}=A{\left[\frac{\eta }{{\left(C_{iv}\right)}^{x}}\right]}^{-B}$$

where initial porosity (*η*) is expressed as a percentage of the volume of voids divided by the total volume of the specimen, while the volumetric cement content (C_iv_) is expressed as a percentage of the volume of cement divided by the total volume of the specimen, and A, *x*, and B are materials-related parameters related to the type of soil and the type of binder as well as their interaction. Constant *A* is expressed in kPa. The accumulated loss of mass (*ALM*) of durability against wetting–drying cycles of improved soil mixtures is also controlled by the porosity-to-binder index, following a power-type relationship as expressed in Equation (2):

(2) $$ALM=A{\left[\frac{\eta }{{\left(C_{iv}\right)}^{x}}\right]}^{B}$$

The index η/(C_iv_) expresses, in a single parameter, the combined influence of both porosity and binder content on the mechanical strength of a material [2,3]. Thus, the relative importance of each factor can be adjusted through the exponent x; that is, when the effect of porosity is more significant, x assumes a value lower than 1.0. For several studies, the R^2^ values obtained for estimating $q_{u}$, $q_{t}$, and *ALM* as functions of the η/C_iv_ ratio are generally high (e.g., [3,4,5]).

Recent studies have increasingly adopted the porosity–binder ratio as a rational framework for correlating the mechanical behavior and durability of stabilized geomaterials. Nierwinski et al. [6] demonstrated that the porosity–cement index effectively predicts both compressive strength and accumulated mass loss in compacted bauxite tailings treated with cement, confirming its applicability to mining residues with variable densities. Similarly, Hanafi et al. [7] reported strong correlations between unconfined compressive strength, initial shear modulus (*G*_o_), and accumulated mass loss (*ALM*) in alluvial clays stabilized with marble dust and cement, where the adjusted porosity index of 0.32 successfully unified the strength and stiffness relationships. Favretto et al. [8] also applied a porosity/lime index to kaolin–bentonite blends, demonstrating that increases in lime content and dry unit weight improved both $q_{u}$ and stiffness, validating the index as a predictive tool for cemented fine-grained soils. These findings reinforce the conclusions of previous works (e.g., [9]), showing that the η/C_iv_ parameter reliably integrates density and binder effects into a single variable that governs cementation efficiency.

Although other approaches have emerged—such as the principal component regression model proposed by Umar et al. [10] for lime and cement-treated clays—these remain complementary rather than substitutive, since they also reveal that porosity and compressibility are the dominant causal mechanisms for strength gain. Additional research, such as Ullas et al. [11] on cement–slag mixtures and Lin et al. [12] on heavy metal sludge ceramic site binders, highlight that the fundamental role of porosity and binder content persists even in alternative cementitious systems, where η/C_iv_ or related indices continue to explain mechanical and microstructural behavior with high statistical reliability (R^2^ > 0.90).

### 1.2. Machine Learning to Predict the Behaviour of Improved Soils

In recent years, machine learning (*ML*) techniques have emerged as a powerful alternative for predicting the mechanical behavior of stabilized soils and artificial geomaterials, offering accuracy and flexibility that surpass traditional empirical correlations, such as the porosity–cement index. Teodoru et al. [13] developed a data-driven framework to predict the unconfined compressive strength ($q_{u}$) of cement-treated clays using 185 experimental data points and multiple regression algorithms, identifying Random Forest as the most accurate model (R^2^ > 0.95). The inclusion of SHAP analysis provided interpretability by confirming cement content and curing time as the most influential variables. Similarly, Noureldin et al. [14] proposed an explainable AI framework for evaluating shear strength in stabilized clays, integrating Shapley values and partial dependence plots to quantify the effects of the water/binder ratio and moisture content, achieving prediction accuracies exceeding 90%. Complementing these efforts, Mohammed et al. [15] applied a suite of ML models to ground granulated blast furnace slag (GGBFS)-stabilized soils, demonstrating that the Extreme Gradient Boosting (XGB) model could explain over 90% of the UCS variance and accurately capture the interactions among curing time, moisture content, and density.

Swamynaidu et al. [16] further confirmed the robustness of ML-based prediction in cement–slag–stabilized clays, with Support Vector Regression (SVR) achieving *R*^2^ value of 0.984 and outperforming conventional regression models. Similarly, Thapa and Ghani [17] employed deep learning architectures (CNN, LSTM, and RNN) to predict $q_{u}$ in nano-silica–stabilized soils, identifying CNN as the most efficient and interpretable model for real-time applications. Kumar et al. [18] extended these approaches to nano-doped fly ash–treated soils, where ensemble models, such as Gradient Boosting Machines (GBM), achieved nearly perfect prediction accuracy (R^2^ = 1.000) and offered physically consistent explanations through SHAP sensitivity analyses. Collectively, these studies demonstrate that machine learning, particularly interpretable models such as Random Forest, XGB, and GBM, can reliably estimate mechanical properties, including $q_{u}$ and shear strength, across diverse soil–binder systems. Moreover, these models provide insight into the relative influence of key geotechnical parameters—binder content, density, curing period, and water ratio—while significantly reducing experimental costs and time.

### 1.3. Motivation and Objective

Based on previous studies, there is currently no evidence in the literature of a comprehensive implementation that correlates and compares the predictive capacity of machine learning (ML) models with the porosity–cement index within a single experimental framework. While the porosity–binder relationship has proven to be a practical empirical approach for estimating the strength and stiffness of cemented soils, and ML techniques have shown excellent potential for predicting complex material behaviors, both approaches have evolved independently so far. Therefore, this study aims to integrate and compare these two methodologies to estimate the unconfined compressive strength ($q_{u}$), the splitting tensile strength ($q_{t}$), and the accumulated mass loss (*ALM*) under wetting–drying cycles of a silty soil improved with Portland cement and recycled ground glass powder (*GGP*). The experimental program includes curing periods of 7, 28, and 90 days, enabling a detailed assessment of the short-, medium-, and long-term mechanical behavior. By developing empirical and data-driven models under identical experimental conditions, this research seeks to determine the most accurate, interpretable, and efficient methodology for predicting the mechanical and durability performance of artificially cemented soils. Ultimately, the findings are expected to contribute to the development of rational, sustainable, and intelligent design strategies for soil stabilization and the creation of advanced geomaterials incorporating industrial by-products.

## 2. Experimental Program

The experimental plan is primarily based on comparing the application of the porosity-to-cement ratio (η/C_iv_) and machine learning methods to estimate the unconfined compressive strength ($q_{u}$), the indirect tensile strength ($q_{t}$), and the durability against wetting–drying cycles through the accumulated mass loss (*ALM*) of a silty soil improved with Portland cement and recycled glass powder.

A total of *n* = 486 tests were performed for the determination of $q_{u}$ and $q_{t}$, and *n* = 81 for *ALM*, as detailed below.

### 2.1. Materials

The materials used in this study included silty soil, high-early-strength Portland cement, and recycled glass powder, along with distilled water used in the preparation of test specimens.

The silty soil was manually collected from a natural slope and exhibited a yellowish color typical of residual deposits in tropical environments. The recycled glass powder (RGP) was obtained from the sedimentation tank of a tempered glass-cutting industry, where the fine particles result from the polishing and cutting process. The Portland cement was obtained from an authorized local distributor and met the requirements for high early-strength cement according to ASTM C150 [19] Type III specifications.

**Figure 1:**
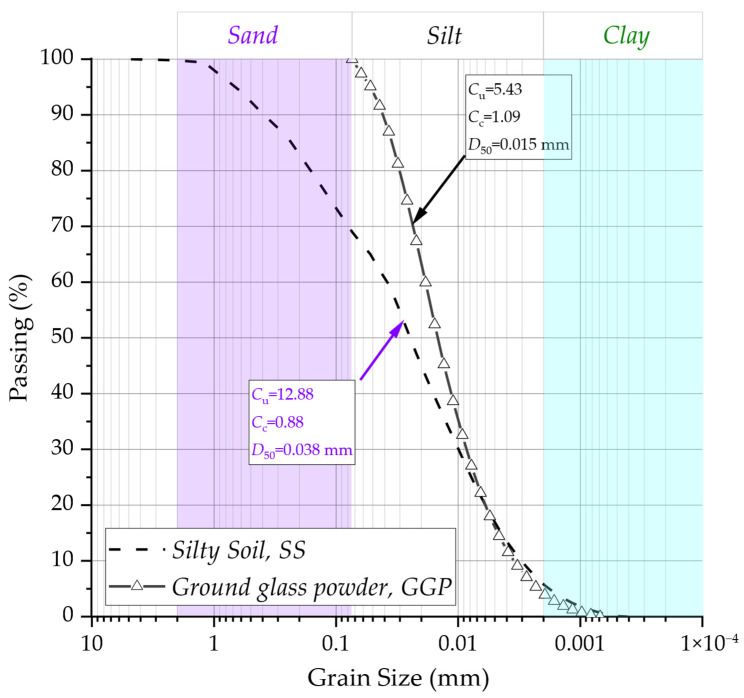
The granulometric curve of the soil sample and ground glass powder *GGP*.

Both the silty soil and the Portland cement were previously characterized by Baldovino et al. [20,21]. Table 1 summarizes the primary geotechnical and physical properties of the materials, as reported in the respective studies. Figure 1 presents the particle size distribution curves of the tested materials, determined by sieve and laser diffraction analyses. The mean particle diameter (D_50_) was determined as 0.015 mm for the ground glass powder (*GGP*) and 0.038 mm for the soil. The coefficients of uniformity (C_u_) were 5.43 for *GGP* and 12.88 for the soil, while the coefficients of curvature (Cc) were 1.09 and 0.88, respectively.

The glass residue used in this study was supplied by a glass processing company located in Curitiba, Brazil. This residue (sludge) originates from the water treatment system required for the grinding and polishing of flat glass. For its use in the soil improvement process, the glass polishing residue was subjected to three preparation stages, resulting in a powdered material referred to as recycled glass powder (*GGP*). The preparation procedures included: (a) drying at 100 ± 5 °C; (b) fragmentation using a Los Angeles abrasion machine for approximately 6–8 h; and (c) sieving through a No. 200 mesh to obtain particles smaller than 0.075 mm (Figure 1). According to Baldovino et al. [21], particles finer than 0.075 mm can act as precursors in geopolymerization processes within soil stabilization mechanisms.

### 2.2. Specimen Molding and Preparation

All specimens, both for compression and tensile tests, were compacted by static pressing in three layers within steel cylindrical molds. The specimens prepared for compression and tensile strength tests had a height of 100 mm and a diameter of 50 mm. For the durability tests, the specimens were compacted to a height of 127 mm and a diameter of 100 mm, in accordance with the ASTM D559 [22] standard for wetting–drying durability evaluation.

After compaction, all specimens were sealed and cured in a humid chamber for 7, 28, and 90 days, prior to conducting the compression and tensile tests. For the durability tests, curing was performed for only 7 days. The details of the curing conditions and mixture compositions are summarized in Table 1.

Table 1 summarizes the experimental matrix adopted for the soil–cement–*GGP* mixtures compacted at three target dry unit weights (13.5 kN/m^3^, 14.5 kN/m^3^, and 15.5 kN/m^3^) and cured for 7, 28, and 90 days. Each mixture was prepared with a fixed soil proportion (100%) and varying cement contents of 3%, 6%, and 9% by dry weight, combined with recycled glass powder (*GGP*) additions of 5%, 15%, and 30%. The selected cement contents (3%, 6%, and 9%) and ground glass powder (GGP) dosages (5%, 15%, and 30%) were defined based on ranges commonly reported in the literature for soil stabilization studies using cement and recycled glass-based binders [23,24,25,26,27]. GGP dosages in the range of 5–30% have been shown to provide meaningful filler and pozzolanic contributions, allowing the evaluation of threshold and saturation effects associated with particle packing and secondary cementitious reactions.

The water content used for molding was maintained at 26% for all specimens. A constant molding water content of 26% was adopted for all mixtures to ensure strict control of experimental variables and to isolate the effects of porosity, cement content, and ground glass powder dosage on mechanical and durability behavior. This water content corresponds approximately to the optimum moisture content of the natural silty soil, allowing the target dry unit weights to be consistently achieved across all mixtures.

For each compaction level and curing period, a total of 27 specimens were prepared and tested. Unconfined compressive strength ($q_{u}$), indirect tensile strength ($q_{t}$), and accumulated loss of mass (*ALM*) due to wetting–drying cycles were evaluated according to the respective test protocols. Durability tests (*ALM*) were conducted only for specimens cured for 7 days, while mechanical strength tests ($q_{u}$ and $q_{t}$) were performed for 7-day, 28-day, and 90-day curing periods.

### 2.3. Unconfined Compressive, Splitting Tensile, and Durability Protocols

The unconfined compression tests were conducted in accordance with ASTM D2166/D2166M–06 [28], while the indirect tensile (Brazilian) tests followed the procedures outlined in ABNT NBR 7222 [24,29]. The durability tests, involving wetting–drying cycles, were performed in accordance with ASTM D559/D559M–15 [22].

All mechanical tests ($q_{u}$ and $q_{t}$) were carried out using a Geotechnik testing machine with a loading rate of 1 mm/min and a maximum capacity of 20 kN. Data acquisition was performed with a force sensitivity of 0.25 N, ensuring precise measurement of load–deformation behavior throughout the tests.

## 3. Machine Learning Analysis

**Figure 2:**
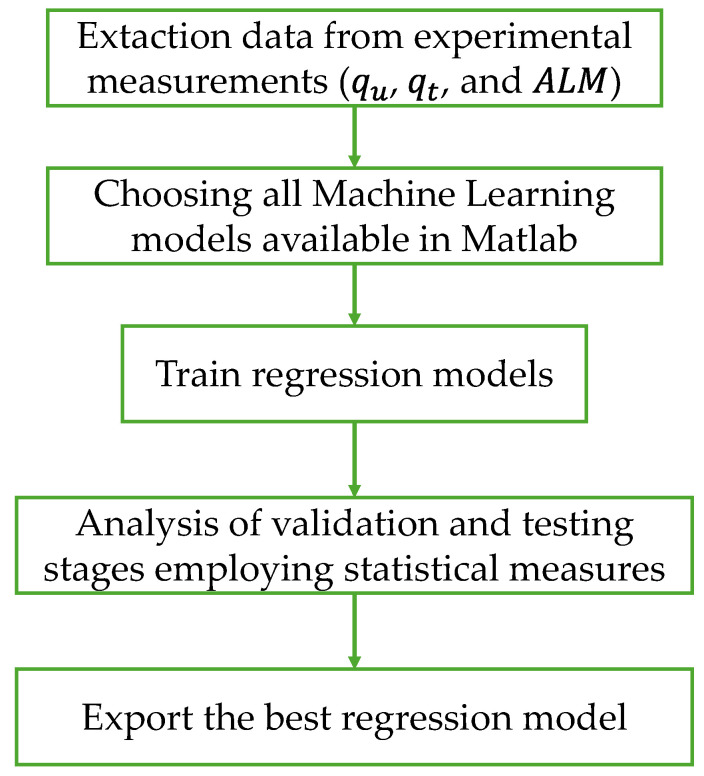
Machine Learning methodology was used in this study.

The machine learning (ML) methodology employed in this research comprises the steps illustrated in Figure 2. Firstly, the dataset was constructed based on the experimental measurements described in Table 1, including all predictors and responses ($q_{u}$, $q_{t}$, and ALM). In total, nine predictors were used for calculation purposes ($T_{C},\;\gamma d,\;T_{V},\;GGP,\;T_{U},\;C_{T},\;G_{s,S},\;G_{s,PG},$ and $G_{s,C}$), where Tc is the curing time, γd is the dry unit weight of blend, Tv is the cement content in percentage, GGP is the ground glass powder, Tu is the water content, Gs,s is the specific gravity of soil, G_s,GGP_ is the specific gravity of GGP, and Gs,c is the specific gravity of cement.

Subsequently, to identify the most suitable ML model, several algorithm types were trained, including linear regression, stepwise linear regression, decision trees, support vector machines, efficient linear models, ensemble models, Gaussian process regression, neural networks, and kernel methods. Each algorithm type, along with its corresponding preset configurations, was trained, validated, and tested. A five-fold cross-validation scheme was employed for validation purposes, with 20% of the data reserved for testing. All ML models were performed using MATLAB R2024b.

As a statistical performance indicator, the coefficient of determination (*R*^2^) was selected. The optimal model was identified as the one exhibiting an *R*^2^ value closest to 1, considering both the validation and testing stages. Finally, the best-performing models for the three responses were exported to the Simulink environment for prediction purposes.

Section 3.1 and Section 3.2 now present the best-performing algorithm employed in this study, as determined by the statistical measures.

### 3.1. Gaussian Process Regression Model

The functions of $q_{u}$ and $q_{t}$ were fitted employing the Gaussian Process Regression (GPR) model. It is a non-parametric kernel-based model that has several subcategories, depending on the definition of the initial parameters, including Squared Exponential, Matern 5/2, Exponential, and Rational Quadratic. In a vector form, the GPR model can be expressed as:

(3) $$P(y|f,\;X)\sim N(y|H\beta +f,\sigma^{2}I)$$

The GPR employs a training set based on predictors ($x_{i}$) and responses ($y_{i}$).

where β = coefficient computed from the dataset, $\sigma^{2}$ = variance, and N corresponds to the Gaussian distribution.

In Equation (3), the following vectors are defined:

(4) $$X=\left(\begin{array}{c} \begin{array}{c} \begin{array}{c} x_{1}^{T}\\ x_{2}^{T}\\ x_{3}^{T} \end{array}\\ \vdots \end{array}\\ x_{n}^{T} \end{array}\right),\;\;\;\;y=\left(\begin{array}{c} \begin{array}{c} \begin{array}{c} y_{1}\\ y_{2}\\ y_{3} \end{array}\\ \vdots \end{array}\\ y_{n} \end{array}\right),\;\;\;\;H=\left(\begin{array}{c} \begin{array}{c} \begin{array}{c} h(x_{1}^{T})\\ h(x_{2}^{T})\\ h(x_{3}^{T}) \end{array}\\ \vdots \end{array}\\ h(x_{n}^{T}) \end{array}\right),\;\;\;\;f=\left(\begin{array}{c} \begin{array}{c} \begin{array}{c} f(x_{1})\\ f(x_{2})\\ f(x_{3}) \end{array}\\ \vdots \end{array}\\ f(x_{n}) \end{array}\right)\;$$

where $f(x_{i})$ corresponds to the latent variables, h(x) is the set of basis functions, n is the samples collected during the experimental program.

The joint distribution can be defined as:

(5) P(y| X)~N(f|0+K(X,X))

The matrix K(X,X) is presented as:

(6) $$K\left(X,X\right)=\left(\begin{array}{ccc} k\left(x_{1},x_{1}\right) & k\left(x_{1},x_{2}\right) & k\left(x_{1},x_{3}\right)\\ k\left(x_{2},x_{1}\right) & k\left(x_{2},x_{2}\right) & k\left(x_{2},x_{3}\right)\\ \vdots & \vdots & \vdots \end{array}\begin{array}{c} \;\;\ldots \;\;\;\\ \ldots \\ \ldots \end{array}\begin{array}{c} k\left(x_{1},x_{n}\right)\\ k\left(x_{2},x_{n}\right)\\ \vdots \end{array}\right)$$

### 3.2. Neural Network

**Figure 3:**
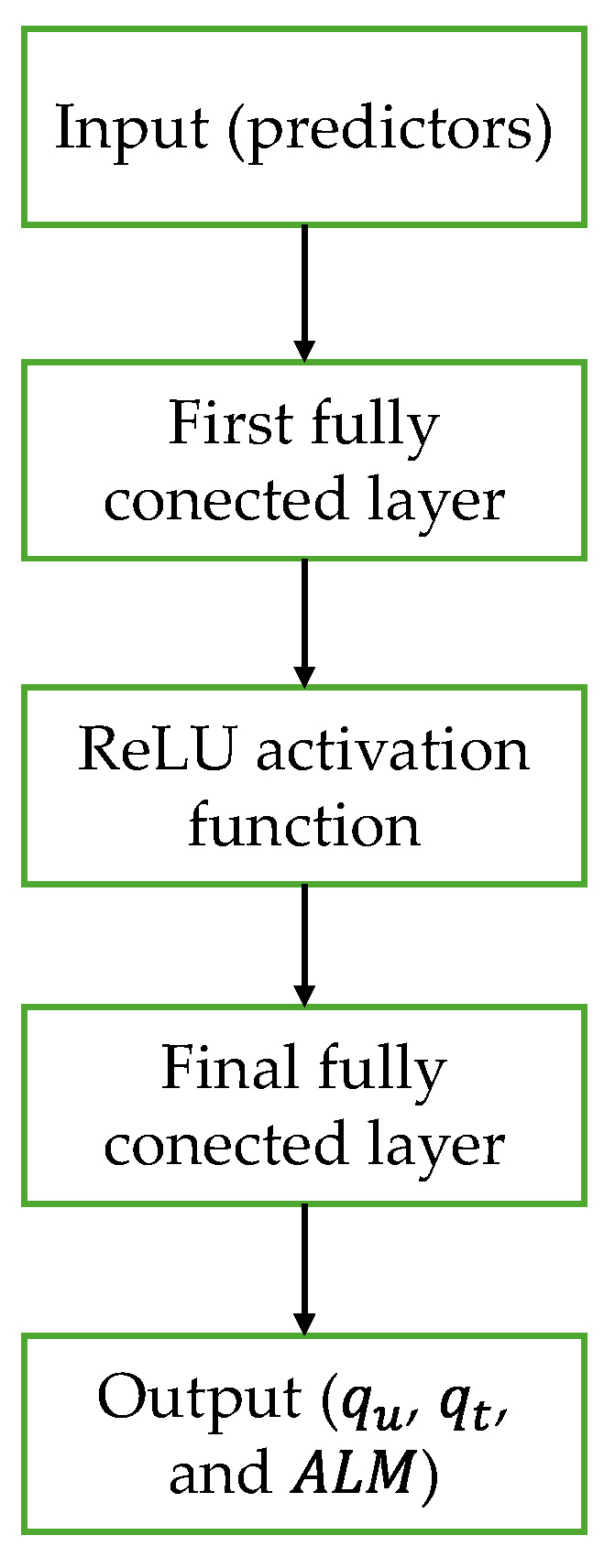
Structure of the neural network.

In this research, different configurations of neural networks were employed. Figure 3 presents the general structure used in this research.

During this research, a three-layer neural network was implemented, comprising three fully connected layers, each containing ten neurons. The rectified linear unit (ReLU) activation function was employed to introduce non-linearity into the model, following this structure:

(7) $$ReLU\left(x\right)=\left\{\begin{array}{c} x,\;\;\;\;x>0\\ 0,\;\;\;\;x\leq 0 \end{array}\right.\;$$

## 4. Results and Discussions

### 4.1. Effects of Porosity-to-Cement Index in Unconfined Compressive Strength of Soil-Cement-Glass Powder Compacted Blends

**Figure 4:**
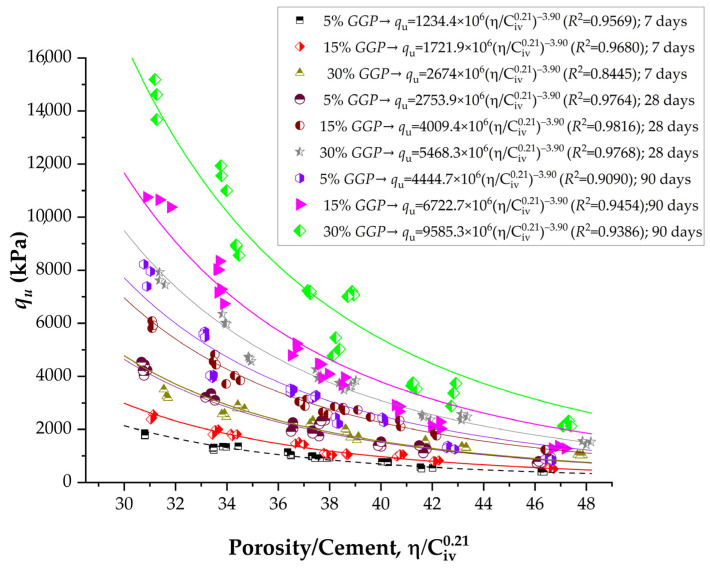
Results of unconfined compressive strength of soil-cement-ground glass powder considering the porosity to cement index (adjusted to 0.21).

Figure 4 illustrates the influence of the porosity–cement ratio on the unconfined compressive strength ($q_{u}$) of soil–cement–*GGP* mixtures at curing periods of 7, 28, and 90 days. The initial porosity of specimens (η) was calculated using:

(8) $$\eta =1-\left[\frac{\gamma_{d}}{1+C+GGP}\right]\left[\frac{1}{\gamma_{S}}+\frac{C}{\gamma_{SC}}+\frac{GGP}{\gamma_{SGGP}}\right]$$

where γ_S_ and γ_GC_ are the specific gravities of the soil and cement, respectively, and γ_SGGP_ is the specific gravity of the glass powder. The volumetric cement content is defined as:

(9) $$C_{iv}=\left[\frac{\gamma_{d}}{1+C+GGP}\right]C$$

where C is the cement content, as expressed in Equations (1), (8) and (9), the relationship used to estimate $q_{u}$ follows the same functional form, where coefficient A is variable, while the exponents *x* and B remain constant. For this type of mixture, *x* = 0.21 and B = −3.90, as determined through iterative mathematical optimization using the Solver tool (Version 2022). In physical terms, the exponent *x* defines the relative sensitivity of the cementation efficiency to volumetric binder content, whereas B captures the rate at which strength decays with increasing porosity-to-binder ratio. Because all mixtures were produced with the same soil, the same cement type, a constant molding water content, and identical specimen geometry and curing conditions, the stress-transfer mechanism remains similar, i.e., particle bonding by cementitious products within a compacted porous skeleton. Changes in GGP content mainly enhance packing and promote secondary cementitious formation, increasing the effective bonding area and matrix continuity; however, they do not alter the fundamental stress path or the functional dependence of strength on η/(C_iv_)*^x^*. Therefore, x and B remain stable, and the long-term hydration/pozzolanic contributions are captured by increases in A rather than changes in the exponents.

**Figure 5:**
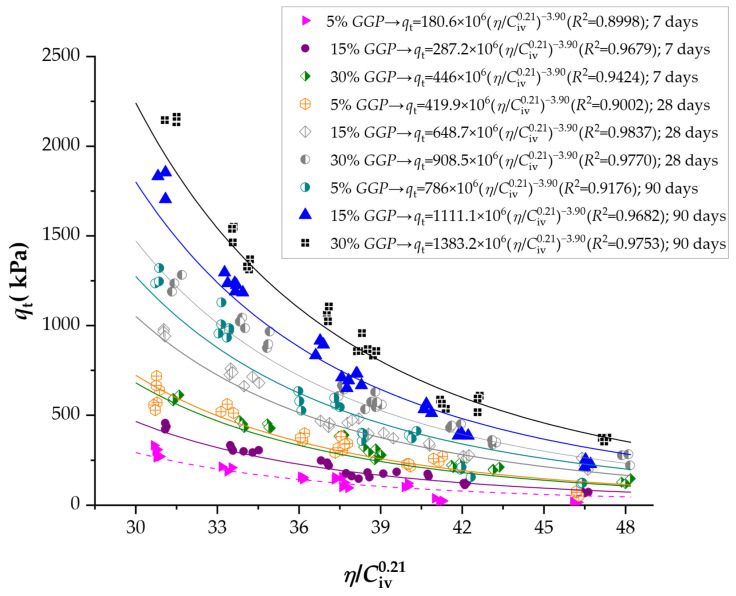
Results of splitting tensile strength of soil-cement-ground glass powder considering the porosity to cement index (adjusted to 0.21).

Figure 5 illustrates the influence of the porosity–cement ratio on the splitting tensile strength ($q_{t}$) of soil–cement–*GGP* mixtures at curing periods of 7, 28, and 90 days. Similarly, for $q_{u}$, splitting tensile values follows the same functional form, where the coefficient A is variable, while the exponents *x* and B remain constant. For this type of test, *x* = 0.21 and B = −3.90.

Table 2 presents the equations controlling the strength of improved soil considering Equation (1) form. The parameter A represents the intrinsic strength potential of each soil–cement–glass powder (*GGP*) mixture and is the only variable factor in the porosity–cement relationship, since the exponents x and B remain constant for all blends (*x* = 0.21 and B = −3.90). Its variation thus expresses the true influence of binder composition and curing time on the mechanical performance of the stabilized soil. In this study, a systematic and consistent increase in A was observed with both curing time and *GGP* content, reflecting the progressive development of the cementitious matrix due to hydration and pozzolanic activity.

The significant strength differences observed between 28 and 90 days of curing, as shown in Figure 4 and Figure 5, indicate that the soil–cement–GGP system had not yet reached mechanical maturity at 28 days. Unlike conventional concrete, where strength gain tends to stabilize after 28 days, cemented soils incorporating recycled glass powder exhibit prolonged strength development due to the slow and sustained pozzolanic reaction of amorphous silica present in the GGP [30]. Between 28 and 90 days, the continued consumption of calcium hydroxide released during cement hydration promotes the formation of secondary cementitious products (e.g., C–S–H and C–A–S–H), leading to further matrix densification and pore refinement [31]. This behavior is reflected by the systematic increase in the strength coefficient A with curing time, confirming that long-term curing plays a critical role in enhancing both compressive and tensile resistance in soil–cement–glass powder geomaterials.

For the unconfined compressive strength ($q_{u}$), A increased progressively from 1234.4 × 10^6^ to 2674 × 10^6^ at 7 days as the GGP content rose from 5% to 30%. This represents an improvement of approximately 117%, indicating that even at early curing ages, the fine glass particles contribute to strength development by filling voids and serving as nucleation sites for C–S–H formation. At 28 days, A values increased sharply to between 2753.9 × 10^6^ and 5468.3 × 10^6^, indicating a near tripling of strength potential compared with the 7-day results. After 90 days of curing, A reached its highest values, ranging from 4444.7 × 10^6^ for the 5% GGP mixture to 9585.3 × 10^6^ for the 30% GGP mixture. The long-term increase in this coefficient, exceeding 250% relative to the initial values, demonstrates the sustained reactivity of the amorphous silica in the recycled glass powder. A similar pattern was observed for the splitting tensile strength ($q_{t}$), with A values increasing from 180.6 × 10^6^ to 446 × 10^6^ at 7 days, from 419.9 × 10^6^ to 908.5 × 10^6^ at 28 days, and reaching 1383.2 × 10^6^ at 90 days for the 30% GGP mixture. The overall increase of nearly sevenfold between 7 and 90 days reflects the progressive enhancement of tensile bonding and interparticle adhesion, driven by the exact mechanisms responsible for the gains in compressive strength.

The coefficient of determination (*R*^2^) values presented in Table 2 demonstrate an excellent statistical correlation between the experimental results and the predictions obtained from the porosity–cement index model. For the unconfined compressive strength ($q_{u}$), the *R*^2^ values ranged from 0.8445 to 0.9816, indicating that the proposed model explains more than 84% to 98% of the experimental variability. Such high coefficients validate the robustness of the porosity–cement approach as a reliable predictor of strength in soil–cement–glass powder (*GGP*) mixtures.

At 28 days, the *R*^2^ values for $q_{u}$ rose to between 0.9764 and 0.9816, indicating a nearly perfect correlation between the measured and estimated values. This improvement aligns with the stage of rapid cement hydration and early pozzolanic activation of the *GGP*, when the microstructure becomes denser and more homogeneous. By 90 days, the R^2^ values ranged from 0.9090 to 0.9454, indicating a slight decrease compared to 28 days. For the splitting tensile strength ($q_{t}$), the correlation coefficients also exhibit high consistency, ranging from 0.8998 to 0.9837. The lower values observed at early ages (7 days) again reflect microstructural immaturity and incomplete formation of interparticle bonds, whereas the highest coefficients at 28 and 90 days correspond to well-developed matrices with strong cementitious and pozzolanic reactions.

### 4.2. Normalization Equations for Estimating the Unconfined Compressive and Splitting Tensile Strength

**Figure 6:**
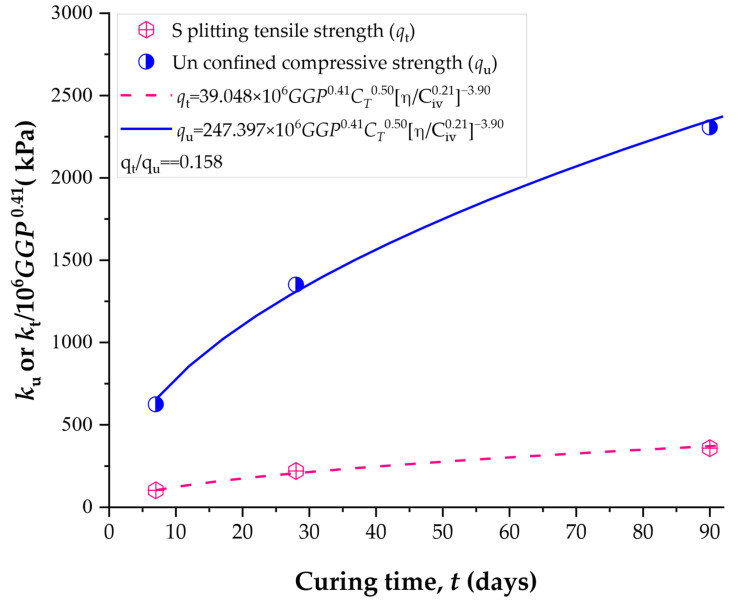
Normalization equations for estimating the strength of soil-cement-GGP compacted blends dependent on curing time, *GGP*, and porosity-to-cement index.

The normalization process begins by dividing the porosity–cement ratio by the corresponding GGP values. These results are then further normalized with respect to the curing time (Tc). In this way, a single equation is developed to estimate both the unconfined compressive strength and the splitting tensile strength results. Thus, Figure 6 presents the normalization of $q_{u}$ and $q_{t}$ results in a unique equation. The equation form is:

(10) $$q_{u\;}and\;q_{t}=k\times {GGP}^{x1}\times {C_{T}}^{x2}{\left[\frac{\eta }{{\left(C_{iv}\right)}^{x}}\right]}^{B}$$

where k, *x*1, and *x*2 are constants. For soil-cement-GGP blends, *x*1 = 0.41 and *x*2 = 0.50. Thus, general equations for estimating the strength of compacted blends are, for $q_{u}$ and $q_{t}$, respectively:

(11) $$q_{u\;}=247.397\times 10^{6}{GGP}^{0.41}\times {C_{T}}^{0.50}{\left[\frac{\eta }{{\left(C_{iv}\right)}^{0.21}}\right]}^{-3.90}$$

(12) $$q_{t\;}=39.048\times 10^{6}{GGP}^{0.41}\times {C_{T}}^{0.50}{\left[\frac{\eta }{{\left(C_{iv}\right)}^{0.21}}\right]}^{-3.90}$$

**Figure 7:**
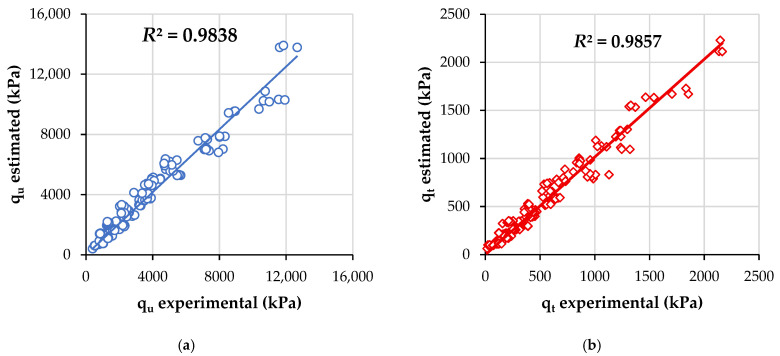
Results of validation of general equations (depending on porosity-cement index) for estimating (**a**) unconfined compressive strength, (**b**) splitting tensile strength.

For validating the equations, experimental values of GGP, time, porosity, and C_iv_ for each specimen were replaced in Equations (11) and (12), for compressive and tensile strength (when corresponding). Thus, Figure 7 provides the *R*^2^ adjusted of estimating equations. Both $q_{u}$ and $q_{t}$ general equations yielded high values of R^2^ (above 0.98), specifically R^2^ = 0.9838 (compressive) and R^2^ = 0.9857 (split tensile).

If Equations (9) and (10) are divided, the value of $q_{t}$/$q_{u}$ = 0.15 is obtained, corresponding to a constant ratio. This indicates that the $q_{t}$ values consistently tend to be approximately 15% of the compressive strength, regardless of the curing time or the amount of added *GGP* and cement. The coefficients A obtained for compressive and splitting tensile strength were compared across all curing periods. The results indicate that the ratio A (for $q_{t}$)/A (for $q_{u}$) remains within a narrow range of approximately 0.14–0.16 for 7, 28, and 90 days of curing, regardless of *GGP* content. This confirms that $q_{t}$ can be considered approximately proportional to $q_{u}$ within the investigated experimental domain, although minor variations with curing time are expected. Therefore, the $q_{t}$/$q_{u}$ = 0.15 ratio should be interpreted as a quasi-constant value rather than a strict universal constant, as presented in other studies [32].

### 4.3. Effects of Porosity-to-Cement Index in Estimating the Durability Against Wetting-Drying Cycles of Compacted Blends

One of the fundamental parameters for assessing the durability of a cemented soil is the accumulated mass loss (*ALM*), which is associated with the reduction in soil mass caused by various degradation processes under wetting–drying cycles. The *ALM* in stabilized soil represents a key indicator of the material’s ability to resist erosion, leaching, and other processes that may compromise its structural integrity over time.

Wetting–drying durability (ALM) was evaluated only for 7-day cured specimens to provide a conservative and sensitive assessment of early-age resistance to degradation, following the ASTM D559 [22] procedure. This design choice enables direct comparison among mixtures under the most critical curing condition where the cementitious matrix is still developing. Because strength and cementation continue to increase with curing time, the 7-day durability results can be interpreted as a lower-bound performance indicator within the investigated mix-design domain.

**Figure 8:**
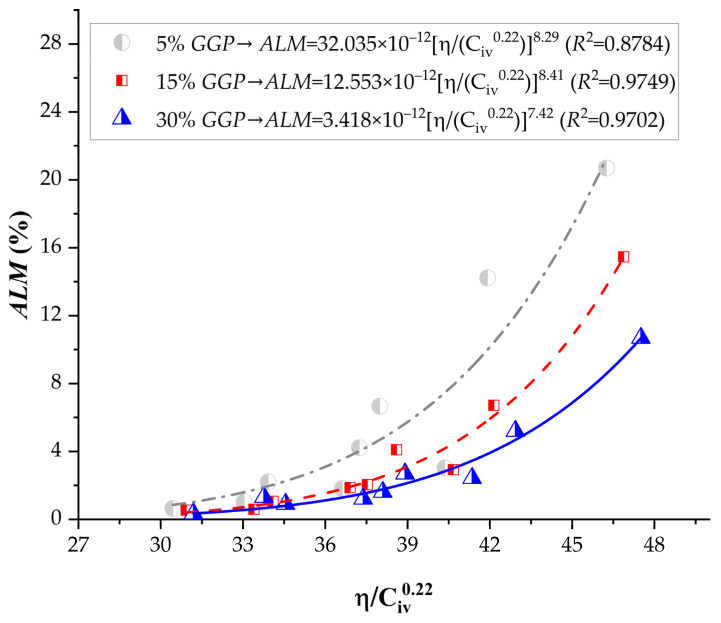
Results of the accumulated loss of mass of soil-cement-ground glass powder, considering the porosity to cement index (adjusted to 0.22).

Figure 8 presents the results of accumulated mass loss (*ALM*) for the soil–cement mixtures compacted at the dry unit weights (Table 1) levels and cured for 7 days, after being subjected to 12 wetting–drying cycles. The accumulated mass loss (ALM) results clearly demonstrate the significant influence of dry density, cement content, and the proportion of recycled glass powder (GGP) on the durability of the stabilized silty soil. As shown in Figure 8, *ALM* decreased consistently as the porosity–binder index $\eta /C_{iv}^{0.22}$ was reduced, confirming that denser mixtures with higher binder content are more resistant to degradation under wetting–drying cycles. Across all data, the evolution of ALM as a function of $\eta /C_{iv}^{0.22}$ follows a clear inverse exponential trend, where small decreases in the index yield significant reductions in mass loss.

Equations controlling ALM, for 5, 15, and 30% GGP are, respectively:

(13) $$ALM=32.035\times 10^{-12}{\left[\frac{\eta }{{\left(C_{iv}\right)}^{0.22}}\right]}^{8.29}\;\;(R^{2}=0.8784)$$

(14) $$ALM=12.553\times 10^{-12}{\left[\frac{\eta }{{\left(C_{iv}\right)}^{0.22}}\right]}^{8.41}(R^{2}=0.9749)$$

(15) $$ALM=3.418\times 10^{-12}{\left[\frac{\eta }{{\left(C_{iv}\right)}^{0.22}}\right]}^{7.42}(R^{2}=0.9702)$$

At a constant *GGP* content of 5%, *ALM* values ranged from 20.69% for the lowest compaction level (γ_d_ = 13.5 kN/m^3^, 3% cement) to 0.65% for the highest compaction and binder content (γ_d_ =15.5 kN/m^3^, 9% cement). This represents an almost 32-fold improvement in durability, highlighting the combined effect of increased density and greater cement availability in forming a more cohesive matrix.

When the *GGP* content increased to 15%, the same tendency was observed, but with generally lower ALM values across all conditions. For instance, at γ_d_ = 13.5 kN/m^3^ and 3% cement, ALM dropped from 20.69% (5% *GGP*) to 15.46% (15% *GGP*), while at γ_d_ = 15.5 kN/m^3^ and 9% cement, the mass loss decreased from 0.65% to 0.54%.

At 30% *GGP*, the *ALM* values became remarkably low for all combinations, particularly at higher dry densities and cement contents. For the most compacted mixture (γ_d_ = 15.5 kN/m^3^, 9% cement), the accumulated mass loss reached only 0.34%, corresponding to the most durable condition in the entire test program. Even at the lowest compaction level (γ_d_ = 13.5 kN/m^3^, 3% cement), ALM was reduced to 10.68%, nearly half the value recorded for the same mixture without GGP (20.69%).

The poorer durability observed in mixtures containing 5% GGP can be attributed to an insufficient filler and pozzolanic contribution to refine the pore network and effectively strengthen particle bonding. At low GGP dosage, pore connectivity remains relatively high, facilitating water ingress/egress during cycling and promoting surface disintegration and mass loss. In contrast, higher GGP contents (15–30%) enhance particle packing and sustain secondary cementitious formation, leading to a denser matrix and improved resistance to wetting–drying-induced degradation.

### 4.4. Machine Learning Results

In total, twenty-eight machine learning (ML) models were trained to identify the most suitable model for each response variable, using the coefficient of determination (*R*^2^) as the statistical performance indicator. For the implementation of the ML presets, a dataset comprising 243 samples was used for $q_{u}$ and $q_{t}$, whereas 81 samples were employed for *ALM*. Appendix A presents the obtained R^2^ values for each algorithm, grouped by model type.

The Gaussian Process Regression (GPR), particularly the Matern 5/2, achieved the best performance for the response variable $q_{u}$, yielding R^2^ values of 0.992 and 0.994 for the validation and testing stages, respectively. Similarly, the exact configuration of GPR produced the most accurate predictions for $q_{t}$, with R^2^ values of 0.988 and 0.987 for validation and testing, respectively. The training times for the machine-learning models were as follows: for $q_{u}$ using the Matern 5/2 GPR, 1.207 s; for $q_{t}$ using the Matern 5/2 GPR, 0.857 s; and for ALM using the Trilayer Neural Network, 3.669 s. The computational costs are notably low, which represents an important advantage for their use in engineering applications.

For the ALM response, the trilayered neural network architecture, comprising three fully connected layers of ten neurons each, provided the best performance, achieving R^2^ values of 0.996 and 0.998 for the validation and testing stages, respectively. In general, both the GPR and neural network algorithms demonstrated strong predictive capability, as evidenced by their consistently high R^2^ values.

In contrast, kernel-based models exhibited comparatively poor performance, showing the lowest R^2^ values among all tested model types.

To prevent overfitting, a cross-validation method was selected in accordance with the dataset’s characteristics. The numerical results obtained during the validation and testing stages demonstrate the adequacy of the machine-learning models.

**Figure 9:**
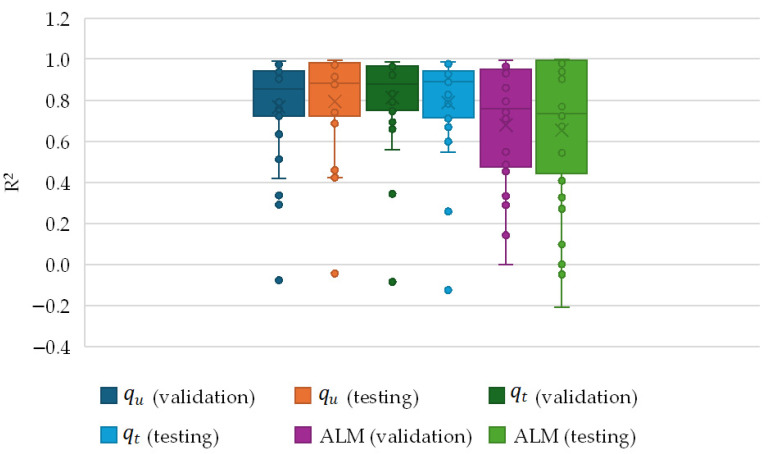
Box-and-whisker plot of the coefficient of determination (R^2^).

A boxplot analysis was conducted to examine the range of R^2^ values obtained from all machine learning (ML) models for the three response variables across both the validation and testing stages. Figure 9 presents the box-and-whisker plots based on the data summarised in Appendix A. In general, the results indicate a consistent trend of satisfactory predictive performance among the ML models, with median R^2^ values exceeding 0.8. The ALM response exhibited the highest median R^2^ values, reaching values close to 1.0 even during the testing stage. Similarly, the $q_{u}$ and $q_{t}$ responses demonstrated strong agreement, characterized by high R^2^ values, despite some variability observed during validation. Several models showed outliers with R^2^ values below 0.4, indicating relatively poor predictive performance. Overall, the majority of ML models yielded excellent agreement between predicted and observed values, confirming their effectiveness as predictive tools.

**Figure 10:**
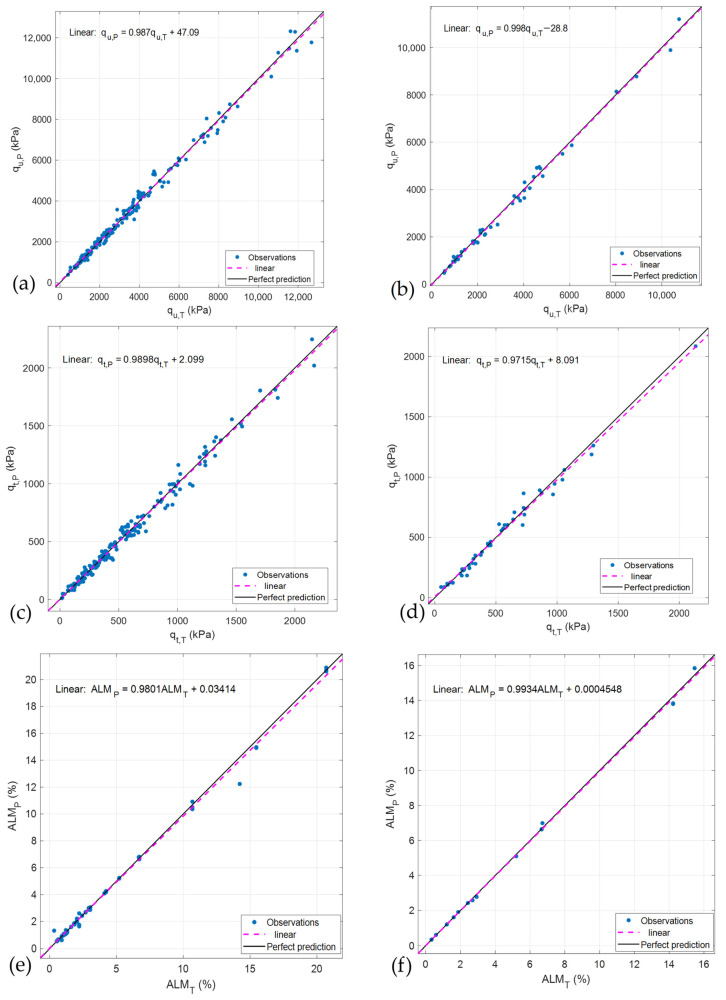
Comparison between predicted and true (experimental characterisation) values of: (**a**) $q_{u}$ for the validation stage; (**b**) $q_{u}$ for testing stage; (**c**) $q_{t}$ for validation stage; (**d**) $q_{t}$ for testing stage; (**e**) ALM for validation stage; and (**f**) ALM for testing stage.

Based on the results presented in Appendix A and Figure 9, the responses $q_{u}$ and $q_{t}$ can be accurately predicted using the Gaussian Process Regression (GPR) model with the Matern 5/2 kernel, while the ALM response is best predicted using the trilayered neural network. Figure 10 illustrates the comparison between the true (experimental) and predicted values obtained from the selected ML models. In this context, the subscript T denotes the true (measured) value, whereas the subscript P represents the predicted value. Table 3 presents the characteristics of the model hyperparameters for the selected machine-learning presets used in this study.

The comparison demonstrates an excellent level of agreement, as the predicted values closely align with the perfect prediction line (black), which is nearly coincident with the dashed magenta line representing the model predictions. In all cases, the experimental data align well with the ideal 1:1 correspondence, confirming the high predictive capability of the developed models. Figure 10a–f present the relationships obtained for the validation and testing stages for each of the responses considered. For example, for $q_{u}$ during the validation stage, the following Equation was calculated:

(16) $$q_{u,P}=0.987q_{u,T}+47.09$$

**Figure 11:**
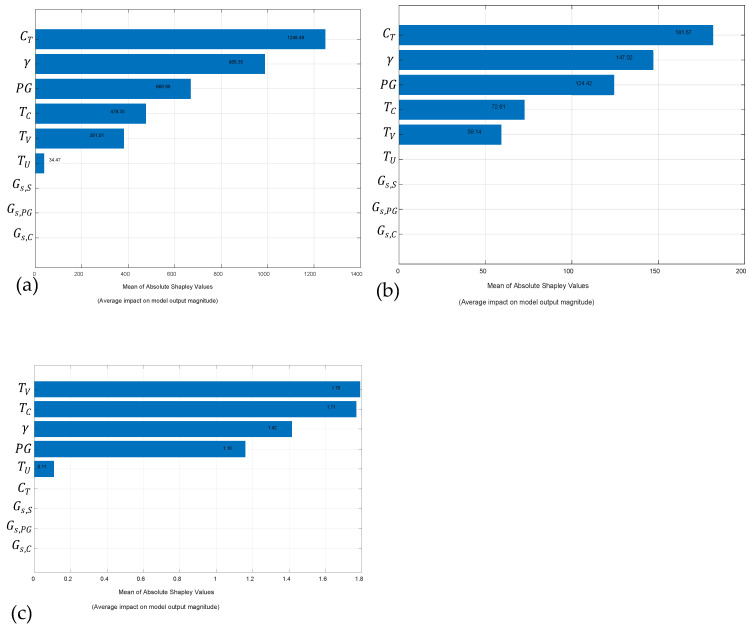
Analysis of shapley values: (**a**) $q_{u}$; (**b**) $q_{t}$; and (**c**) ALM.

The Shapley values were computed to identify the most influential predictors for representing the responses $q_{u}$, $q_{t}$, and ALM. Figure 11 presents the results, indicating that the predictors $C_{T}$, γ, $T_{V}$, PG, $T_{U}$, and $T_{C}$ are the most relevant for $q_{u}$ and $q_{t}$, with curing time ($C_{T}$) emerging as the most significant factor (see Figure 11a,b). The remaining predictors exhibit minimal influence on these responses. In the case of the ALM response, the most relevant predictors are $T_{C}$, γ, $T_{V}$, PG, and $T_{U}$ (see Figure 11c).

A comparison was conducted between the porosity–binder ratio and the machine learning presets, as summarized in Table 4. The results confirm that ML techniques provide a reliable approach for predicting $q_{u}$, $q_{t}$, and ALM, as evidenced by the high coefficients of determination (R^2^) obtained. These values were even slightly higher than those computed using the porosity–binder ratio equations, demonstrating the superior predictive capability of the ML models.

The empirical equations derived from the η/C_iv_ index exhibited excellent performance for both $q_{u}$ and $q_{t}$, with R^2^ values of 0.9838 and 0.9857, respectively—confirming the robustness, consistency, and physical interpretability of the porosity–cement framework. However, the ML models outperformed the empirical formulations in all cases. The Gaussian Process Regression with a Matern 5/2 kernel achieved superior accuracy, reaching R^2^ values above 0.992 for both validation and testing in predicting $q_{u}$ and $q_{t}$, indicating its ability to capture nonlinear interactions in the dataset that are not fully represented in the porosity-based Equation. The contrast becomes even more evident in durability predictions: while the porosity–binder equations for *ALM* showed moderate-to-high correlations (R^2^ = 0.8784–0.9749 depending on *GGP* content), the trilayer neural network achieved nearly perfect predictive performance (R^2^ = 0.996–0.998). These findings suggest that ALM, a degradation parameter influenced by multiple interactive mechanisms, benefits substantially from the multidimensional learning capacity of ML architectures. Although ML models offer superior accuracy, the porosity–binder ratio remains an indispensable tool due to its interpretability and mechanistic insight, indicating that both approaches are complementary in advancing the predictive understanding of cemented geomaterials. For ALM, the values of B range from 7.42 to 8.29, while the exponent x remains constant at 0.22. The constant A varies from 3.418 × 10^−12^ to 32.035 × 10^−12^ (see Table 4). In contrast, the trilayered neural networks exhibit considerably less variability, with SHAP values ranging from 0.11 to 1.79 (see Figure 11).

The mechanistic behaviour captured by the η/Civ framework is adequately represented using the parameters obtained from the experimental programme. Specifically, for $q_{u}$ these include $C_{T}$, γ, PG, $T_{C}$, $T_{V}$, and $T_{U}$; for $q_{t}$, $C_{T}$, γ, PG, $T_{C}$, and $T_{V}$; and for ALM, $T_{V}$, $T_{C}$ γ, PG, and $T_{U}$.

It is of the utmost importance to emphasise that machine-learning methods can serve as a complementary tool, enabling rapid decision-making in practical engineering applications. The selected machine-learning models may be applied within the range covered by the experimental programme. This implies that exporting the machine-learning model to the Simulink environment is sufficient to quantify the values of $q_{u}$, $q_{t}$, and ALM, using only the data obtained from the experimental programme.

## 5. Conclusions

- This study demonstrated that the porosity–cement index (η/C_iv_) provides a robust and physically meaningful framework for correlating the mechanical behavior of soil–cement–ground glass powder (*GGP*) geomaterials. High coefficients of determination (R^2^ > 0.95) were obtained for both un:s of 7, 28, and 90 days, confirming the reliability of the index for strength prediction.
- The strength evolution was governed primarily by the intrinsic strength coefficient A, which increased systematically with curing time and GGP content, while the exponents *x* = 0.21 and *B*= −3.90 remained constant. At 90 days, mixtures containing 30% GGP achieved increases in $q_{u}$ exceeding 250% and in $q_{t}$ approaching 700% compared with early-age values, reflecting sustained hydration and long-term pozzolanic reactions associated with the amorphous silica in recycled glass powder.
- Durability performance, evaluated through accumulated mass loss (*ALM*) under wetting–drying cycles at 7 days of curing, was strongly controlled by the porosity–binder index. Denser mixtures with higher cement and *GGP* contents exhibited significantly enhanced resistance to degradation, with *ALM* values as low as 0.34%. The 7-day durability assessment provided a conservative indicator of early-age performance, highlighting the beneficial role of *GGP* in refining pore structure and improving matrix cohesion.
- The implementation of machine learning techniques proved highly effective for predicting $q_{u}$, $q_{t}$, and *ALM.* Gaussian Process Regression with a Matern 5/2 kernel yielded the most accurate predictions for strength parameters, achieving *R*^2^ values above 0.99 in both validation and testing stages, while a trilayered neural network provided near-perfect prediction of ALM (*R*^2^ = 0.998). SHAP analysis confirmed curing time, density, cement content, and GGP dosage as the most influential predictors, ensuring model interpretability.
- A direct comparison between empirical η/C_iv_-based equations and machine learning models revealed that, although ML approaches provide superior predictive accuracy, the porosity–cement index remains indispensable due to its physical interpretability and simplicity. The complementary use of both methodologies enables rational mix design and rapid performance prediction of cemented soils incorporating recycled glass powder.
- The proposed experimental–analytical framework contributes to the development of sustainable and intelligent soil stabilization strategies by integrating physically based indices with data-driven models. The findings support the effective reuse of recycled glass powder as a supplementary binder, providing a solid basis for future extensions to other soil types, various curing conditions, and long-term durability assessments.
- From a practical engineering perspective, the results indicate that cement contents of 6–9% combined with GGP dosages of 15–30% and adequate compaction levels offer an optimal balance between strength, durability, and material efficiency for soil stabilization applications. The η/C_iv_ framework provides a rational tool for preliminary mix design, while the trained ML models enable rapid performance prediction and decision-making in real-world projects within the range investigated.
- The proposed interpretations of strength and durability evolution are primarily supported by macroscopic mechanical and durability tests; therefore, future research should incorporate microstructural analyses (e.g., SEM–EDS, XRD, or pore size distribution) to further validate the cementation and pozzolanic mechanisms inferred from the porosity–cement index.

## Figures and Tables

**Table 1:** Mixed proportion design for soil-cement-GGP compacted blends and cured under 7, 28, and 90 days.

Molding *γ*_d_ (kN/m^3^)	Soil (%)	Cement (%)	Glass Powder (%)	Water Content (%)	Curing Times (Days)	Specimens by Test	Test	Total Specimen
13.5	100	3, 6, and 9	5, 15, and 30	26	7	27	$q_{u}$$,\;q_{t}$, and *ALM*	81
	100	3, 6, and 9	5, 15, and 30	26	28	27	$q_{u}$ $\;\mathrm{and}\;q_{t}$	54
	100	3, 6, and 9	5, 15, and 30	26	90	27	$q_{u}$ $\;\mathrm{and}\;q_{t}$	54
14.5	100	3, 6, and 9	5, 15, and 30	26	7	27	$q_{u}$$,\;q_{t}$, and *ALM*	81
	100	3, 6, and 9	5, 15, and 30	26	28	27	$q_{u}$ $\;\mathrm{and}\;q_{t}$	54
	100	3, 6, and 9	5, 15, and 30	26	90	27	$q_{u}$ $\;\mathrm{and}\;q_{t}$	54
15.5	100	3, 6, and 9	5, 15, and 30	26	7	27	$q_{u}$$,\;q_{t}$, and *ALM*	81
	100	3, 6, and 9	5, 15, and 30	26	28	27	$q_{u}$ $\;\mathrm{and}\;q_{t}$	54
	100	3, 6, and 9	5, 15, and 30	26	90	27	$q_{u}$ $\;\mathrm{and}\;q_{t}$	54

**Table 2:** Results of equations for estimating unconfined compressive and splitting tensile strength of soil-cement-*GGP* compacted blends, derivation of porosity-to-cement ratio index.

Curing Time (Days)	Test	*GGP* (%)	A Value (×10^6^)	*x* Exponent	B Exponent	*R* ^2^
7	$q_{u}$	5	1234.4	0.21	−3.90	0.9569
		15	1721.9	0.21	−3.90	0.9680
		30	2674	0.21	−3.90	0.8445
28		5	2753.9	0.21	−3.90	0.9764
		15	4009.4	0.21	−3.90	0.9816
		30	5468.3	0.21	−3.90	0.9768
90		5	4444.7	0.21	−3.90	0.9090
		15	6722.7	0.21	−3.90	0.9454
		30	9585.3	0.21	−3.90	0.9386
7	$q_{t}$	5	180.6	0.21	−3.90	0.8998
		15	287.2	0.21	−3.90	0.9679
		30	446	0.21	−3.90	0.9424
28		5	419.9	0.21	−3.90	0.9002
		15	648.7	0.21	−3.90	0.9837
		30	908.5	0.21	−3.90	0.9770
90		5	786	0.21	−3.90	0.9176
		15	1111.1	0.21	−3.90	0.9682
		30	1383.2	0.21	−3.90	0.9753

**Table 3:** Characteristics of the model hyperparameters for the selected ML presets.

Model	Matern 5/2 GPR	Trilayer Neural Network
Basis function	Constant	—
Kernel type	Isotropic Matern 5/2	—
Kernel scale	Automatic	—
Signal standard deviation	Automatic	—
Sigma	Automatic	—
Standardisation of data	Yes	Yes
Optimisation of numerical parameters	Yes	—
Number of fully connected layers	—	3
Neurons per layer	—	10
Activation function	—	ReLU
Maximum iterations	—	1000
Regularisation strength	—	0

**Table 4:** Statistical comparison between porosity binder ratio and machine learning models.

Response	Porosity-Binder Ratio		Machine Learning		
	Equation	*R* ^2^	Selected Preset	R^2^	
				Validation	Testing
$q_{u}$	$247.397\times 10^{6}{GGP}^{0.41}{C_{T}}^{0.50}{\left[\frac{\eta }{{\left(C_{iv}\right)}^{0.21}}\right]}^{-3.90}$	0.9838	Matern 5/2 GPR	0.992	0.994
$q_{t}$	$39.048\times 10^{6}{GGP}^{0.41}{C_{T}}^{0.50}{\left[\frac{\eta }{{\left(C_{iv}\right)}^{0.21}}\right]}^{-3.90}$	0.9857	Matern 5/2 GPR	0.988	0.987
ALM	$32.035\times 10^{-12}{\left[\frac{\eta }{{\left(C_{iv}\right)}^{0.22}}\right]}^{8.29}$	0.8784	Trilayered Neural Network	0.996	0.998
	$12.553\times 10^{-12}{\left[\frac{\eta }{{\left(C_{iv}\right)}^{0.22}}\right]}^{8.41}$	0.9749			
	$3.418\times 10^{-12}{\left[\frac{\eta }{{\left(C_{iv}\right)}^{0.22}}\right]}^{7.42}$	0.9702			

## Data Availability

The original contributions presented in this study are included in the article. Further inquiries can be directed to the corresponding authors.

## Appendix A

**Table A1:** Computation of R^2^ for all machine learning algorithms.

Model Type	Preset	$q_{u}$		$q_{t}$		ALM	
		Validation	Test	Validation	Test	Validation	Test
Linear Regression	Linear	0.757	0.771	0.807	0.732	0.552	0.558
	Interactions Linear	0.920	0.942	0.946	0.919	0.888	0.939
	Robust Linear	0.723	0.741	0.798	0.737	0.334	0.098
Stepwise Linear Regression		0.92	0.916	0.942	0.943	0.916	0.860
Tree (T)	Fine T	0.791	0.876	0.835	0.783	0.795	0.724
	Medium T	0.512	0.718	0.680	0.598	0.453	0.565
	Coarse T	0.291	0.424	0.344	0.258	0.000	−0.049
Support Vector Machine (SVM)	Linear SVM	0.722	0.747	0.792	0.729	0.470	0.271
	Quadratic SVM	0.904	0.914	0.964	0.902	0.710	0.946
	Cubic SVM	0.336	0.423	0.746	0.669	0.977	0.769
	Fine Gaussian SVM	0.773	0.881	0.831	0.891	0.486	0.561
	Medium Gaussian SVM	0.941	0.972	0.953	0.926	0.548	0.408
	Coarse Gaussian SVM	0.634	0.686	0.694	0.680	0.158	0.001
Efficient Linear	Efficient Linear Least Squares	0.727	0.741	0.766	0.711	0.550	0.673
	Efficient Linear SVM	0.419	0.459	0.557	0.546	0.289	0.326
Ensemble	Boosted Trees	0.912	0.936	0.925	0.927	0.742	0.747
	Bagged Trees	0.782	0.887	0.837	0.826	0.556	0.545
Gaussian Process Regression (GPR)	Squared Exponential GPR	0.991	0.993	0.982	0.986	0.930	0.999
	Matern 5/2 GPR	0.992	0.994	0.988	0.987	0.946	0.999
	Exponential GPR	0.987	0.995	0.987	0.987	0.952	0.998
	Rational Quadratic GPR	0.991	0.994	0.987	0.987	0.943	0.999
Neural Network (NN)	Narrow NN	0.906	0.926	0.947	0.897	0.988	0.995
	Medium NN	0.946	0.880	0.968	0.977	0.994	0.995
	Wide NN	0.975	0.994	0.984	0.988	0.982	0.997
	Bilayered NN	0.938	0.987	0.923	0.889	0.966	0.979
	Trilayered NN	0.963	0.992	0.967	0.947	0.996	0.998
Kernel	SVM Kernel	−0.077	−0.044	−0.085	−0.124	0.142	−0.208
	Least Squares Regression	0.808	0.469	0.660	0.805	0.772	0.559

Note: the grey cells represent the best model.
